# Hyperbolastic growth models: theory and application

**DOI:** 10.1186/1742-4682-2-14

**Published:** 2005-03-30

**Authors:** Mohammad Tabatabai, David Keith Williams, Zoran Bursac

**Affiliations:** 1 Department of Mathematical Sciences, Cameron University, 2800 W Gore Blvd., Lawton, OK 73505, USA; 2 Department of Biostatistics, University of Arkansas for Medical Sciences, Slot 820, Little Rock, AR 72205, USA

**Keywords:** Hyperbolastic models

## Abstract

**Background:**

Mathematical models describing growth kinetics are very important for predicting many biological phenomena such as tumor volume, speed of disease progression, and determination of an optimal radiation and/or chemotherapy schedule. Growth models such as logistic, Gompertz, Richards, and Weibull have been extensively studied and applied to a wide range of medical and biological studies. We introduce a class of three and four parameter models called "hyperbolastic models" for accurately predicting and analyzing self-limited growth behavior that occurs e.g. in tumors. To illustrate the application and utility of these models and to gain a more complete understanding of them, we apply them to two sets of data considered in previously published literature.

**Results:**

The results indicate that volumetric tumor growth follows the principle of hyperbolastic growth model type III, and in both applications at least one of the newly proposed models provides a better fit to the data than the classical models used for comparison.

**Conclusion:**

We have developed a new family of growth models that predict the volumetric growth behavior of multicellular tumor spheroids with a high degree of accuracy. We strongly believe that the family of hyperbolastic models can be a valuable predictive tool in many areas of biomedical and epidemiological research such as cancer or stem cell growth and infectious disease outbreaks.

## 1. Introduction

The analysis of growth is an important component of many clinical and biological studies. The evolution of such mathematical functions as Gompertz, logistic, Richards, Weibull and Von Bertalanffy to describe population growth clearly indicates how this field has developed over the years. These models have proved useful for a wide range of growth curves [1]. In the logistic model, the growth curve is symmetric around the point of maximum growth rate and has equal periods of slow and fast growth. In contrast, the Gompertz model does not incorporate the symmetry restriction and has a shorter period of fast growth. Both the logistic and Gompertz have points of inflection that are always at a fixed proportion of their asymptotic population values. A number of recent publications have utilized some of these models. Kansal [2] developed a cellular automation model of proliferative brain tumor growth. This model is able to simulate Gompertzian tumor growth over nearly three orders of magnitude in radius using only four microscopic parameters. Brisbin [3] observed that the description of alligator growth by fixed-shape sigmoid models such as logistic, Gompertz or Von Bertalanffy curves may not be adequate because of the failure of the assumption that a constant curve shape holds across treatment groups. There are many applications of Gompertz, logistic and Von Bertalanffy models to multicellular tumor spheroid (MTS) growth curves [4-9]. Yin [10] introduced the beta growth function for determinate growth and compared it to the logistic, Gompertz, Weibull and Richards models. He showed that the beta function shares several characteristics with the four classic models, but was more suitable for accurate estimation of final biomass and duration of growth. Ricklef [18] investigated the biological implications of the Weibull and Gompertz models of aging. Castro [19] studied a Gompertzian model for cell growth as a function of phenotype using six human tumor cell lines. They concluded that cell growth kinetics can be a phenotypic organization of attached cells. West [20,21] introduced an ontogenetic theory of growth, which is based on first principles of energy conservation and allocation. A review of these studies reveals that the sigmoid character of the classical three or more parameter growth functions, such as the logistic or Von Bertalanffy, may not adequately fit three-dimensional tumor cell cultures, which often show complex growth patterns. Models that have been found to provide the best fit were modified or generalized versions of the Gompertz or logistic functions. The 1949 data on the polio epidemic [11] provide another classic example of a situation in which none of the above models fit the data very well. Our purpose is to introduce three new growth models that have flexible inflection points and can fit data with different shapes. We apply our proposed models to the 1949 polio epidemic data [11] and Deisboeck's MTS volume data [9] and compare their fit with four classical models: logistic [12], Richards [13], Gompertz [14] and Weibull [15].

## 2. The Hyperbolastic Model H1

First, we start by considering the following growth curve, which produces flexible asymmetric curves through nonlinear ordinary differential equations of the form



or



with initial condition

*P*(*t*_0_) = *P*_0_

where *P*(*t*) represents the population size at time *t*, *β *is the parameter representing the intrinsic growth rate, *θ *is a parameter, and *M *represents the maximum sustainable population (carrying capacity), which is assumed to be constant, though in general the carrying capacity may change over time. For growth curves, *β *has to be positive, leading to an eventually increasing curve with an asymptote at *M*; *β *can be negative only for eventual inhibition curves or decay profiles. We refer to growth rate model (1) as the hyperbolastic differential equation of type I. If *θ *= 0, then the model (1) reduces to a logistic differential equation and equation (2) reduces to a general logistic model [12]. Solving the equation (1) for the population *P *gives



where



and arcsinh(t) is the inverse hyperbolic sine function of *t*. We call the function *P*(*t*) in equation (2) the hyperbolastic growth model of type I or simply H1. To reduce the number of parameters, observed values of *P*_0 _and *t*_0 _are used to obtain an approximate value of *α*. Notice that the asymptotic value of *P*(*t*) is



From equation (1) we calculate the second derivative



If we set *θ *= 0, then the second derivative



when



In other words, when the population *P *reaches half the carrying capacity *M*, the growth  is most rapid and then starts to diminish toward zero. If we assume *θ *≠ 0, then the growth  is most rapid at the time t*, such that t* satisfies the following equation



If the carrying capacity changes at discrete phases of a hyperbolastic growth, then a bi-hyperbolastic or multi-hyperbolastic model may be appropriate.

## 3. The Hyperbolastic Model H2

Now we consider an alternative growth curve through a nonlinear hyperbolastic differential equation of the form



with initial condition *P*(*t*_0_) = *P*_0 _and *γ *> 0, where tanh stands for hyperbolic tangent function, *M *is the carrying capacity, and *β *and *γ *are parameters. As in the H1 model, parameter *β *has to be positive for increasing growth curves with an asymptote at *M *and is negative only for decay profiles. We refer to the growth rate model (3) as the hyperbolastic differential equation of type II.

Solving equation (3) for population size *P *gives the three parameter model



where



We call the function *P*(*t*) in equation (4) the hyperbolastic growth model of type II or simply H2. As in the H1 model, observed values of *P*_0 _and *t*_0 _are used to obtain an approximate value of *α *and to reduce the number of parameters.

Notice from equation (4) that for positive values of *β*, *P*(t) approaches *M *as *t *tends to infinity and for negative values of *β*, *P*(*t*) approaches zero as *t *tends to infinity. Moreover, from equation (3), we calculate the second derivative



where csch and coth represent hyperbolic cosecant and hyperbolic cotangent, respectively. The growth rate  is most rapid at time t* provided that *t *= t* satisfies the following equation



If *γ *= 1, then the growth rate  is most rapid at time *t *= t* if the following equality is true



## 4. The Hyperbolastic Model H3

Finally, we consider a third growth curve through the following nonlinear hyperbolastic differential equation of the form



with initial condition *P*(*t*_0_) = *P*_0 _where M is the carrying capacity and *β*, *γ *and *θ *are parameters. We refer to model (5) as the hyperbolastic ordinary differential equation of type III.

The solution to equation (5) is a four parameter model

*P*(*t*) = *M *- *α EXP*[-*β t*^*γ *^- arcsinh(*θ t*)]     (6)

where

*α *= (*M *- *P*_0_) *EXP*[*β t*_0_^*γ *^+ arcsinh(*θ t*_0_)].

We call the function *P*(*t*) in equation (6) the hyperbolastic growth model of type III or simply H3. If *θ *= 0, then this model reduces to the Weibull function [15]. The growth rate  is most rapid at time t* such that



If we define the *a*(*t*) as the rate of generation of new tumor cells and *b*(*t*) as the rate of loss of tumor cells, for instance, then

 and .

The growth rate can then be written as



If no tumor cells are lost (*b*(*t*) = 0), the tumor size *P*(*t*) follows the equation

*P*(*t*) = *M *[*β t*^*γ *^- arcsinh(*θ t*)].

## 5. Application of Hyperbolastic Models

### Statistical Analysis

We analyze two data sets by fitting the general logistic model of the form

[12], where



the Richards model of the form

[13], where



the Gompertz model of the form

*P*(*t*) = *M EXP*[-*α EXP*(-*M β t*)] [14], where



the Weibull model of the form

*P*(*t*) = *M *- *α EXP*(-*β t*^*γ*^) [15], where

*α *= (*M *- *P*_0_) *EXP*(*β t*_0_^*γ*^)

and the hyperbolastic models H1, H2, and H3 described above. Obviously some of these models are closely related. Nonetheless, the parameter values may be quite different when these models are fitted to a single set of data. The logistic model used here is a two parameter symmetric model, while the Richards model generalizes the logistic model by introducing an additional parameter (*γ*) to the equation to deal with asymmetrical growth. The Richards function reduces to the logistic equation if *γ *= 1. The Gompertz equation, which is a two parameter asymmetric equation, attains its maximum growth rate at an earlier time than the logistic. In the Weibull equation, *β *and *γ *are constants defining the shape of the response. In all seven models *M *is a constant, the maximum value or the upper asymptote, which is estimated by non-linear regression. In each instance we express one model parameter (*α*) as the function of the other parameters and initial observed value *P*_0 _at time *t*_0_, which allows us to reduce the number of parameters to be estimated and also anchors the first predicted value to the original value observed at the initial time point.

The mean squared error (MSE) and the *R*^2 ^value from the nonlinear regression, as well as the absolute value of the relative error (RE), which was defined as



were used to indicate the prediction accuracy or goodness of fit for all seven fitted models. All models were fitted using SAS v.9.1 PROC NLIN (SAS Institute Inc., Cary, NC) and SPSS v.12.0.1 (SPSS Inc., Chicago, IL). The best fitting functions and their derivatives were plotted using Mathematica v.4.2 (Wolfram Research Inc., Champaign, IL) to find the growth rates and accelerations.

### Analysis of the Polio Epidemic Data

**Figure 1:**
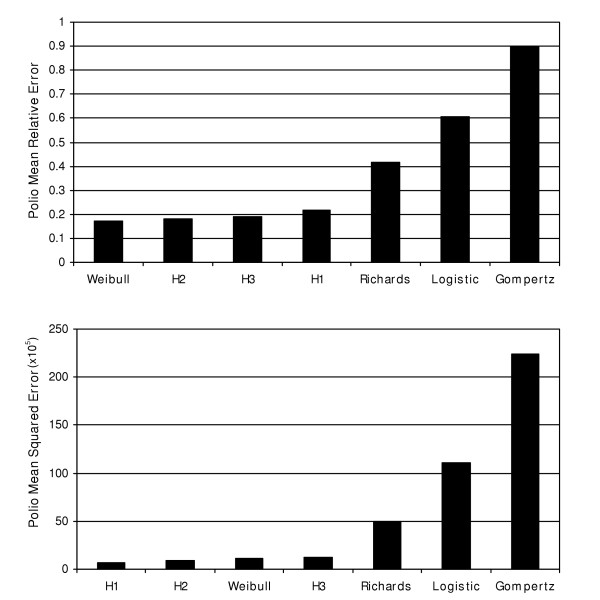
Bar graphs represent mean(s) of the relative error(s) and mean squared error for the polio models.

In 1949, the United States experienced the second worst polio epidemic in its history. Table 1 gives the cumulative number or incidence of polio cases diagnosed on a monthly basis [11] and the number of cases predicted by each of the seven models. The data originally appeared in the 1949 Twelfth Annual Report of the National Foundation for Infantile Paralysis. Absolute values of RE, MSE and *R*^2 ^for the seven tested models are given in Table 2. Average RE and MSE plots for the seven polio epidemic models are graphically presented in Figure 1.

**Table 1:** Number of observed and predicted polio cases using seven models.

*Month*	*Polio Cases*	*H1*	*H2*	*Weibull*	*H3*	*Richards*	*Logistic*	*Gompertz*
0	494	494	494	494	494	494	494	494
1	759	242.47	544.99	494.15	467.57	838.76	901.13	1112.71
2	1016	278.44	720.94	505.28	452.96	1424.12	1632.93	2224.48
3	1215	526.24	1153.82	635.10	563.29	2418.00	2924.19	4016.77
4	1619	1279.87	2218.78	1334.67	1262.75	4105.48	5130.07	6649.96
5	2964	3506.99	4917.77	3769.25	3730.08	6970.11	8697.85	10223.41
6	8489	9510.12	11444.30	9859.95	9872.33	11824.88	13987.47	14754.76
7	22377	21110.00	23267.60	20665.19	20687.08	19913.74	20898.37	20177.15
8	32618	33011.70	34655.40	32802.34	32777.73	31513.43	28575.53	26352.24
9	38153	39160.25	39855.70	39775.43	39753.72	39660.86	35708.48	33093.26
10	41462	41203.34	41247.00	41264.73	41277.59	41382.42	41316.48	40190.97
11	42375	41763.32	41522.90	41339.95	41358.99	41573.16	45174.27	47437.25

**Table 2:** Absolute value of the relative error(s), MSE and *R*^2 ^for seven tested models with polio data.

*Month*	*RE(H1)*	*RE(H2)*	*RE(W)*	*RE(H3)*	*RE(R)*	*RE(L)*	*RE(G)*
0	0.00	0.00	0.00	0.00	0.00	0.00	0.00
1	0.68	0.28	0.35	0.38	0.11	0.19	0.47
2	0.73	0.29	0.50	0.55	0.40	0.61	1.19
3	0.57	0.05	0.48	0.54	0.99	1.41	2.31
4	0.21	0.37	0.18	0.22	1.54	2.17	3.11
5	0.18	0.66	0.27	0.26	1.35	1.93	2.45
6	0.12	0.35	0.16	0.16	0.39	0.65	0.74
7	0.06	0.04	0.08	0.08	0.11	0.07	0.10
8	0.01	0.06	0.01	0.01	0.03	0.12	0.19
9	0.03	0.05	0.04	0.04	0.04	0.06	0.13
10	0.01	0.01	0.01	0.00	0.00	0.00	0.03
11	0.01	0.02	0.02	0.02	0.02	0.07	0.12

*MSE*	6.61 × 10^5^	8.72 × 10^5^	11.09 × 10^5^	12.45 × 10^5^	50.21 × 10^5^	111.12 × 10^5^	223.65 × 10^5^

*R*^2^	0.9983	0.9978	0.9969	0.9969	0.9864	0.9667	0.9336

**Figure 2:**
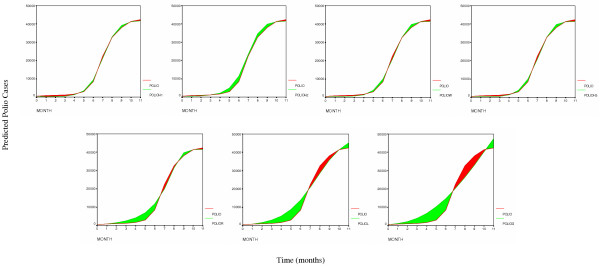
Area represents the error between the observed and predicted polio cases for the seven tested models in the following order starting from top left: H1, H2, Weibull, H3, Richards, logistic and Gompertz.

**Figure 3:**
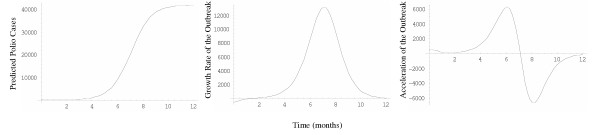
Curves represent a) predicted number of polio cases using best fitting H2 model b) first derivative of the previous function or the growth rate of the polio outbreak and c) second derivative or acceleration of the polio outbreak.

The results show that the H1  and H2  models provide the best fit to the polio incidence data, followed by Weibull  and H3  models. The Richards , logistic  and Gompertz  models are clearly inadequate to describe the polio incidence growth pattern (Figures 1 and 2). The second derivative of the fitted H1 function suggests that the highest incidence of the polio epidemic cases occurred between July and August of 1949 (Figure 3).

### Analysis of the MTS Growth Data

**Figure 4:**
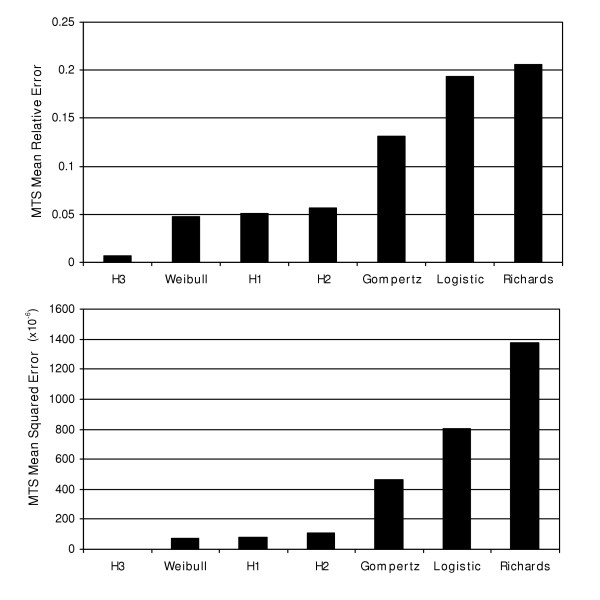
Bar graphs represent mean(s) of the relative error(s) and mean squared error for the MTS volume growth models.

In 2001, Deisboeck et al. [9] studied the development of multicellular tumor spheroids (MTS) by creating a microtumor model. They claimed that a highly malignant brain tumor is an opportunistic, self-organizing and adaptive complex dynamic bio-system rather than an unorganized cell mass. Mature MTS possess a well-defined structure, comprising a central core of dead cells surrounded by a layer of non-proliferating, quiescent cells, with proliferating cells restricted to the outer, nutrient-rich layer of the tumor. Angiogenesis is a process by which new blood vessels are created from existing ones. A cell, which would be malignant, detaches from the tumor and uses the new blood supply to travel throughout the body. These authors suggested that such growth can be described by both the Gompertz and logistic functions. Using Deisboeck's MTS with "heterotype attractor" data, the four classical models were compared with the hyperbolastic ones to identify which model predicted the MTS volume most accurately. The observed and predicted MTS volume values are presented in Table 3. The absolute values of RE, MSE and *R*^2 ^for each model are given in Table 4. The average RE and MSE plots for the seven cancer volume models are graphically presented in Figure 4.

**Table 3:** Observed and predicted MTS volume using seven models.

*Time(hr)*	*Volume*	*H3*	*Weibull*	*H1*	*H2*	*Gompertz*	*Logistic*	*Richards*
0	0.087	0.087	0.087	0.087	0.087	0.087	0.087	0.087
24	0.080	0.080	0.088	0.067	0.089	0.099	0.107	0.108
48	0.082	0.083	0.096	0.093	0.099	0.116	0.132	0.134
72	0.129	0.127	0.125	0.133	0.125	0.140	0.162	0.165
96	0.188	0.189	0.184	0.186	0.182	0.177	0.200	0.202
120	0.255	0.256	0.259	0.251	0.261	0.234	0.245	0.245
144	0.318	0.317	0.317	0.320	0.316	0.327	0.302	0.297

**Table 4:** Absolute value of the relative error(s), MSE and *R*^2 ^for seven tested models with MTS volume data.

*Time(hr)*	*REH3*	*REW*	*REH1*	*REH2*	*REG*	*REL*	*RER*
0	0.000	0.000	0.000	0.000	0.000	0.000	0.000
24	0.002	0.096	0.166	0.109	0.239	0.339	0.353
48	0.016	0.168	0.132	0.201	0.416	0.607	0.634
72	0.019	0.030	0.029	0.028	0.089	0.257	0.277
96	0.007	0.020	0.009	0.034	0.059	0.061	0.072
120	0.002	0.016	0.016	0.022	0.083	0.038	0.038
144	0.002	0.004	0.007	0.006	0.030	0.052	0.066

*MSE*	3.33 × 10^-6^	74.21 × 10^-6^	82.83 × 10^-6^	109.2 × 10^-6^	464.35 × 10^-6^	802.06 × 10^-6^	1374.42 × 10^-6^

*R*^2^	0.9998	0.9974	0.9974	0.9957	0.9808	0.9100	0.8972

**Figure 5:**
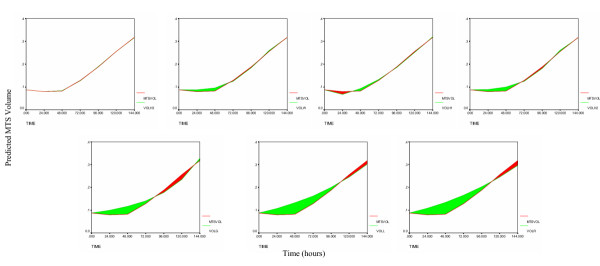
Area represents the error between the observed and predicted MTS volume for the seven tested models in the following order starting from top left: H3, Weibull, H1, H2, Gompertz, logistic and Richards.

**Figure 6:**
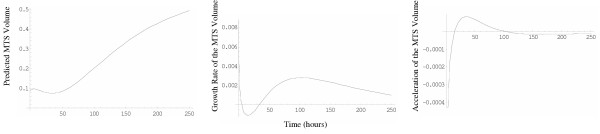
Curves represent a) predicted MTS volume using best fitting H3 model b) first derivative of the previous function or the growth rate of the MTS volume and c) second derivative or the acceleration of the MTS volume growth.

The results indicate that the H3 model  has superior prediction accuracy for this particular data set. It is followed by the Weibull , H1  and H2  models, which predict with similar accuracy. Finally, the Gompertz , logistic  and Richards  models resulted in the least precise fit of the seven (Figures 4 and 5). Even though the Weibull model was the second best, the mean relative error associated with it was almost seven times the mean relative error for the best-fitting H3 model. Over 144 hours, MTS growth follows decelerating growth dynamics with some shrinking during early stages (Figure 6). The first derivative of *P*(*t*) (growth rate) indicates that the MTS volume growth rate is zero at t = 4.90 hours and t = 34.27 hours (Figure 6). The second derivative of the fitted H3 function shows that the acceleration is slowest at t = 15.27 hours and fastest when t = 103.03 hours (Figure 6).

**Figure 7:**
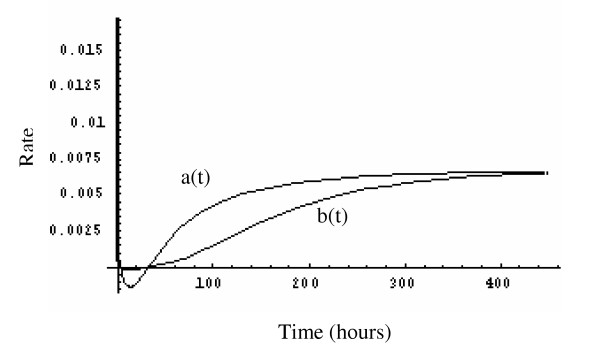
Functions represent the rate of generation of new tumor cells a(t) and rate of loss of tumor cells b(t).

Figure 7 compares the MTS rate of generation of new tumor cells (*a*(*t*)) to the rate of loss of tumor cells (*b*(*t*)). One can clearly see that the gap between the two rates becomes smaller and gradually approaches zero. Notice that as the gap approaches zero, the growth rate  also approaches zero.

## 6. Discussion

Obviously no model can accurately describe every biological phenomenon that researchers encounter in their practice and the same is true for our models. Many models have been developed to deal with sigmoid growth [16] and new ones are continuously being proposed. The logistic function is symmetric around the point of inflection. The Richards function is more flexible and can fit asymmetric growth patterns [10,17]; however, it has more parameters than the logistic function. The Gompertz function has the same number of parameters as the logistic function and the Weibull function has the same number of parameters as the Richards function and both can fit asymmetric growth, but they are not very flexible [10].

**Figure 8:**
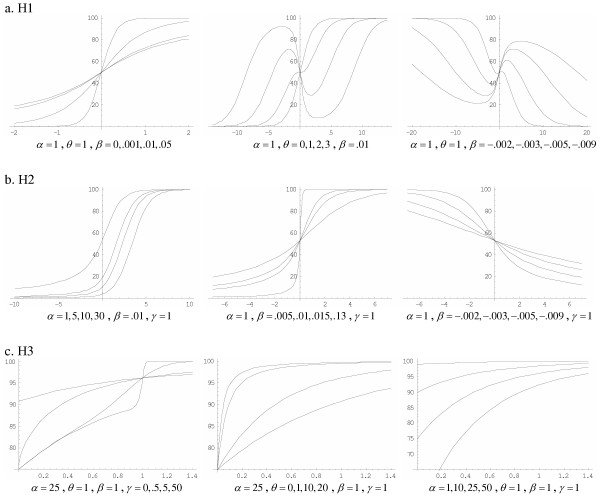
Functions illustrate the flexibility of the H1, H2 and H3 models. One parameter is varied while the others are held constant to demonstrate the capability of the models to fit different growth or decay patterns. In all examples parameter M is held constant at 100.

The H1 function has one more parameter than the logistic and Gompertz functions, but it is more flexible and can fit asymmetric growth patterns as well as increasing and decreasing growth, as shown in the MTS volume example. The H2 function has the same number of parameters as H1 and can fit asymmetric curves, but it cannot fit decreasing growth patterns, so it is less flexible. The H3 function has the same flexibility as the H1 function at the expense of one more parameter, similar to the Weibull and Richards equations. Some of the flexibility of the H1, H2 and H3 functions is illustrated in Figure 8.

The logistic and Gompertz functions have two parameters that are easily interpretable. Like Yin [10], we encountered problems in trying to provide initial parameter values in the Weibull function. One can arrive at a satisfactory solution by trial and error, or using a grid search in SAS PROC NLIN by providing a range of starting values. These functions can be easily implemented in SPSS or SAS PROC NLIN (see Additional file 1) or other readily available software packages. Non-linear function parameters that have biological meaning are more advantageous for statistical parameterization of such equations. The same can be said for some of the parameters in the three proposed models, which can be determined by summarizing the data or using the above suggestions. Table 5 provides estimates for the parameters of the H1, H2 and H3 models. If necessary, an additional parameter called the shift parameter may be added to a model to improve the fit of the data to a model.

**Table 5:** Parameter estimates (with standard errors in parentheses) for H1, H2 and H3 models applied in two examples.

*Model*	*Parameter*	*Polio Estimate*	*MTS Estimate*
H1	M	41951.4 (603.8)	0.5633 (0.16)
	*β*	3.9 × 10^-5 ^(3.06 × 10^-6^	0.0395 (0.02)
	*θ*	-2.6851 (0.29)	-0.2171 (0.05)
			
			
H2	M	41574.9 (670.6)	0.3360 (0.03)
	*β*	2.9 × 10^-6 ^(7.02 × 10^-7^)	1.9 × 10^-5 ^(4.7 × 10^-5^)
	*γ*	1.8865 (0.13)	2.6784 (0.55)
			
			
H3	M	41359.6 (817.5)	0.5871 (0.02)
	*β*	4.11 × 10^-6 ^(5.62 × 10^-6^)	0.0371 (0.01)
	*γ*	6.18 (0.67)	0.8575 (0.05)
	*θ*	-0.00065 (0.01)	-0.0256 (0.003)

While the results presented are valid only for the data sets used in this study, these models can have much wider application than shown here. We successfully applied them to several other data sets including craniofacial and stem cell growth data and the results indicate supreme prediction accuracy for the hyperbolastic models. Based on the results presented in this paper and others not shown here, we can say that the H3 model performs the best with cancer cell, craniofacial and stem cell growth data. However, it is reasonable to compare models for fit before deciding on the selection of the "best" one. With appropriate parameter adjustments in H1 or H2, one can derive regression type models for dichotomous or polytomous response variables, and use these models in survival data problems, reliability studies, business applications and many other situations.

Finally, our hyperbolastic models show very promising results. In both the above discussed data sets, they fitted the data with smaller MSE, smaller mean RE and higher prediction accuracy than the logistic, Richards and Gompertz, which were the worst fit models in both cases. Our models are accurate and simple and two of them generalize the logistic and Weibull models. They can be easily implemented and tested in readily available software packages or routines. We strongly believe that choosing a flexible and highly accurate predictive model such as hyperbolastic can significantly improve the outcome of a study and it is the accuracy of a model that determines its utility. We strongly recommend usage of such models to the scientific community and practitioners and urge comparison of them with classical models before decisions on model selection are made.

## Competing interests

The author(s) declare that they have no competing interests.

## Authors' contributions

MT carried out the mathematical derivations, programming and testing of the models and the drafting and reviewing of the manuscript. DKW was involved in verifying the mathematical derivations, programming the models and reviewing the manuscript. ZB participated in the derivations, verification and formatting of the functions, programming and testing of the models and writing the manuscript.

## Supplementary Material

Additional File 1 SAS code used to fit H1, H2 and H3 models to MTS volume data. Click here for file
