# Supplementary material for: Hyperbolastic growth models: theory and application
Source: Theor Biol Med Model. 2005 Mar 30;2:14. doi: 10.1186/1742-4682-2-14 (PMC1084364; doi:10.1186/1742-4682-2-14)
Supplement: Additional File 1 — SAS code used to fit H1, H2 and H3 models to MTS volume data. [file 1742-4682-2-14-S1.doc]

**Appendix**

SAS code used to fit H1, H2 and H3 models for MTS volume growth.

**data** mtsvolume;

input hour volume;

cards;

0 0.087

24 0.080

48 0.082

72 0.129

96 0.188

120 0.255

144 0.318

;

**run**;

/* H1 */

title 'H1 model';

**proc** **nlin** data=mtsvolume method=marquardt noitprint maxiter=**100**;

parms

m=**0** to **1** by **0.1**

beta=**0.0001**

theta=**0** to **10** by **1**;

model volume=m/(**1**+(m-**0.087**)/**0.087***exp(**0***m*beta+theta*log(**0**+sqrt(**1**)))

*exp((-m*beta*hour)-theta*log(hour+sqrt(hour****2**+**1**))));

output out=h1mtsout p=h1pred;

**run**;

/* H2 */

title 'H2 model';

**proc** **nlin** data=mtsvolume method=marquardt noitprint maxiter=**100**;

parms

m=**0** to **1** by **0.1**

beta=**0.000001**

theta=**0** to **10** by **1**;

model volume=m/(**1**+(m-**0.087**)/(**0.087***log(**1**+sqrt(**2**)))

*log(exp(-m*beta*hour**theta)+sqrt(exp(-**2***(m*beta*hour**theta))+**1**)));

output out=h2mtsout p=h2pred;

**run**;

/* H3 */

title 'H3 model';

**proc** **nlin** data=mtsvolume method=marquardt noitprint maxiter=**100**;

parms

m=**0.5**

beta=**0.037**

gama=**0.8575**

theta=-**0.0255**;

model volume=m-(m-**0.087**)*exp(-beta*hour**gama-log(theta*hour+sqrt(**1**+(theta*hour)****2**)));

output out=h3mtsout p=h3pred;

**run**;
